# The One Giant Leap commercial wireless power meter can be used for sprint kayaking with the appropriate calibration

**DOI:** 10.3389/fphys.2025.1461644

**Published:** 2025-08-07

**Authors:** Joshua A. Goreham, Michel Ladouceur

**Affiliations:** Biodynamics, Ergonomics, Neuroscience (BENLab) Laboratory, Division of Kinesiology, School of Health and Human Performance, Faculty of Health, Dalhousie University, Halifax, NS, Canada

**Keywords:** power, force, elite, female, athletes, on-water

## Abstract

**Purpose:**

Two experiments were conducted to determine the construct and concurrent validity of a commercial kayak paddle shaft power meter (OGL) for measuring force and power output in female sprint kayakers.

**Methods and Results:**

Construct validity: Seven female participants used the same OGL paddle to complete 30 s trials at different stroke rates (60, 80, 100, maximum strokes per minute) while a global positioning system measured kayak velocity. Regression analysis provided a large coefficient of determination (R^2^≥0.83) between mean power and mean velocity (f(x) = 6.892 × 3). Concurrent validity: Two known weight combinations were used to calibrate the paddle (wide range: 51.5–394.9 N; narrow range: 100.6–247.7 N), whereas both left and right sides of the shaft were statically loaded eight separate times with known weights (51.5 N–394.9 N at 49.1 N intervals) to test its concurrent validity. The right side of the shaft had proportional bias (p < 0.001) and the left side of the shaft had fixed bias (65.7 ± 21.1 N, p = 0.017) when calibrated with a narrow range. Neither shaft side had proportional bias, but both shaft sides had small, fixed biases (left: 18.3 ± 7.4 N, p = 0.043; right: 9.3 ± 3.0 N, p = 0.018) when calibrated with a wide range.

**Conclusion:**

The study establishes that even though the OGL reports power values that appear to have construct validity up to 4.6 m s^-1^, calibration with a range of weights that encompasses the projected applied forces is needed to improve the accuracy of the force measurement, and thus the power calculation, by the OGL.

## 1 Introduction

As innovative technology becomes available, on-water measurement of paddle forces and power are becoming popular in sprint kayaking. These measurements are highly beneficial to performance evaluation because they quantify the mechanical workload required to be successful, while other external variables may be affected by changes in the environment (i.e., velocity, stroke rate (SR), etc.) (Hogan et al., 2020a). Power output is often measured in other cyclical sports; however, it remains uncommon in on-water sprint kayaking even though average power output is related to an increase in sprint kayaking performance on a kayak ergometer (Bishop et al., 2002; Michael et al., 2008; 2009), most probably because of the higher complexity of the paddling movement (McDonnell et al., 2013). However, there is a need to measure power output to help coaches and athletes determine workload while training. Researchers and practitioners have been searching for a tool to measure on-water propulsive forces from a kayak paddle since at least the 1980s (Aitken and Neal, 1992; Bonaiuto et al., 2020; Gomes et al., 2015; Kong et al., 2020; MacDermid and Fink, 2017; Romagnoli et al., 2022; Stothart et al., 1986a; Stothart et al., 1986b). There have been many iterations of instrumented paddles and power meters, but no single paddle is widely accepted as a gold standard. One study suggested that a lack of products available with an “acceptable level of validity”, below 5% measurement error (Brosnan et al., 2021; Crang et al., 2021), was the primary reason (McDonnell et al., 2013). For the interpretations of values from a measurement system, and inferences based on these measurements to be meaningful, it is critical that the evaluation measures demonstrate acceptable validity and reliability. There are several types of measurement validity, and this research focuses on construct and concurrent validity. Establishing the degree to which a measure assesses the hypothetical construct it is intended to reflect is central to construct validity. Whereas, comparing the measured values to a known “gold standard” is the tenet of concurrent validity.

Multiple recent studies have used a specific power meter (Kayak Meter Pro, One Giant Leap (OGL), Nelson, NZ) to measure the propulsive force and power of a sprint kayak stroke (Hogan et al., 2020a; Hogan et al., 2020b; Hogan et al., 2021; Kong et al., 2020; Macdermid et al., 2019; Winchcombe et al., 2019). The OGL paddle has six strain gauges and an inertial measurement unit, which calculate force output and power using proprietary algorithms (Winchcombe et al., 2019). The exact use of the paddle varies between studies, but it is commonly used to monitor training load, physiological testing (Hogan et al., 2021; 2020b; 2020a; Macdermid et al., 2019; Winchcombe et al., 2019), and/or kayak stroke kinetics (Kong et al., 2020). Macdermid and Fink (2017) established the construct validity of the OGL paddle by comparing its measurements to the cubic relationship between power output and velocity in aquatic locomotion (Barbosa et al., 2010; Di Prampero et al., 1974; Michael et al., 2009). This relationship can be explained further by reducing the equation of power. To increase kayak velocity, the kayaker must overcome the hydrodynamic drag forces resisting the athlete kayak system; therefore, increasing the overall power output. Since power is equal to force multiplied by velocity, we can substitute drag force into the equation. Drag force (DF) is equal to Equation 1,

DF=12ρAKv2 (1)

where ρ is equal to water density, A is kayak surface area, K is drag coefficient and v is velocity. By multiplying both sides of the equation by v, power (P) becomes Equation 2,

P=12ρAKv3 (2)

Which makes it proportional to velocity cubed (Macdermid and Fink, 2017).

Unfortunately, the study looked at the power meters in slalom kayak training, and thus may not be transferable to elite level sprint kayaking (Hogan et al., 2020a; Hogan et al., 2020b; Hogan et al., 2021; Kong et al., 2020; Winchcombe et al., 2019). For example, the low on-water paddling velocities collected during their validation (i.e., maximum velocity of 2.49 m s^-1^) are well below that of race velocities for female 200 m sprint kayakers (4.95 ± 0.46 m s^-1^) (Goreham et al., 2021). Furthermore, the study used a narrow range of known forces during their experiment, with only three known weights tested to a maximum of 155.9 N. This amount of force is significantly lower than the mean peak forces applied to the water by elite sprint kayakers at velocities of 4.14 ± 0.25 m s^-1^ (301.1 ± 23.1 N) (Bonaiuto et al., 2020).

The purpose of this study was to determine the OGL power meter measurement validity for on-water sprint kayak. The first experiment extended the construct validity of the OGL power meter by including velocities that are comparable to levels found in sprint kayak. It was hypothesized that the OGL paddle’s mean power output would have a strong cubic relationship with mean kayak velocity. If found to have acceptable construct validity, a second experiment determined the concurrent validity of the paddle force measurements. It was hypothesized that the OGL paddle’s force outputs would not be significantly different from applied known weight forces. Finally, a supplementary data acquisition was carried out to determine if a wider range of calibration weights would provide better concurrent validity than the suggested range of calibration weights.

## 2 Materials and methods

### 2.1 Construct validity

#### 2.1.1 Participants

Seven elite (Canadian national level and above) female sprint kayak athletes (21.6 ± 4.6 years old, 1.69 ± 0.04 m, 66.8 ± 5.4 kg, 12.7 ± 5.1 years of kayaking experience) participated in the construct validity portion of the study. All participants consented to participating in the study in accordance with Dalhousie University’s Research Ethics Board (No. 2020-5127).

#### 2.1.2 Experimental protocol

Data were collected on a marked 1,000 m sprint kayak racecourse with participants using their personal kayaks and a short, stiff OGL power meter (Gen 2.1) with Brača IV (765) blades. Prior to testing the distances between the blade tips and middle knuckles of each hand, blade tip to blade tip, blade tip to shaft datums (marks provided by the manufacturer on the paddle shaft), the blade twist, and the blade type were recorded in the OGL web application. The calibration process consisted of three steps. The first step recorded the output of the load cells during an unloaded condition (leaning vertically against a wall). For the second step, the paddle was placed horizontally on two thin (width: 0.3 m) support surface, one located in the middle of the right blade and the second located at the approximate location of the top hand (left hand, Figure 2A). Known weights (i.e., 100.6 N and 247.7 N) were hung at the approximate location of the bottom hand (right hand) as per the manufacturer’s guidelines and load cell outputs were recorded. The third step consisted in replicating the second step using the left blade and location of the right hand for the location of the fulcrums and the location of the left hand for the location of the calibration weights. The recorded data was used to generate the OGL power meter load cells scale factors.

**FIGURE 1:**
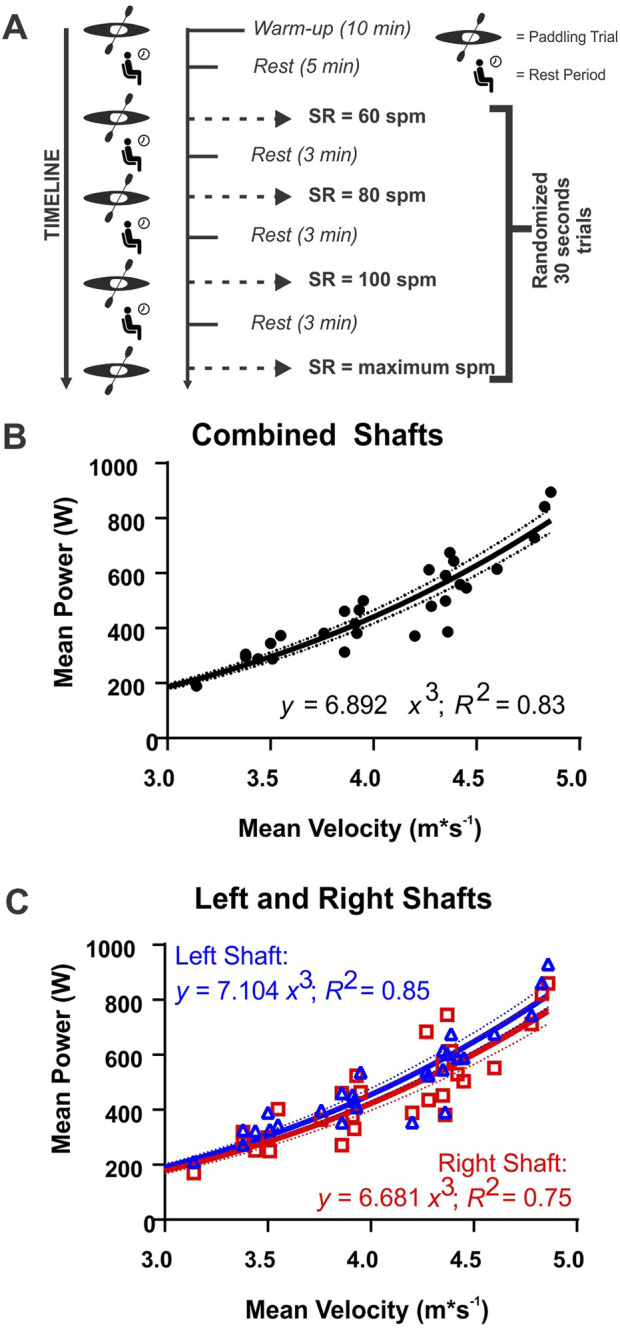
**(A)** The experimental protocol for the construct validation portion of the study. SR, stroke rate; spm, strokes per minute; min, minutes. Mean velocity vs mean power outputs measured from **(B)** the average of all ten strokes (circles) and **(C)** the right (squares) and left (triangles) shaft sides, separately. Red and blue lines indicate the cubic function’s line of best fit for the right and left sides of the shaft, respectively. Dotted lines indicate 95% confidence bands. *R* ^2^, coefficient of determination; W, watts; m•s^-1^, meters per second.

The experimental protocol began with a 10 min, individual-led warm-up, followed by a 5-min rest period. The participant then completed four, 30-s trials. One for each of four different SRs (random order: 60 strokes per minute (spm), 80 spm, 100 spm, and maximum spm), with a 3-min rest period between trials (Figure 1A). These SRs were selected as they are often used in training (60 spm, 80 spm) and in competition (100 spm, maximum spm). Participants started the trial from a static position and were instructed to increase their SR slowly until they reached the intended trial SR (within 10 s). The average SR during the final 20 s of the trial was required to be within ±5 strokes per minute of the intended SR to be analyzed.

All data were collected in calm environmental conditions (15.8°C ± 3.5°C air temperature, 14.3°C ± 2.1°C water temperature, 0.73 ± 0.51 m s^-1^ tail wind). Data collection in similar environmental conditions reduced the effect of wind and water temperature on the variability of the measurement properties. OGL paddle data were collected using a Samsung Galaxy Tab S2 tablet with OGL’s web based software. Force and power output from the paddle was measured at 50 Hz during each stroke’s water phase. Kayak velocity data were collected for each trial using an inertial measurement unit (IMU; LMS330DL, STMicroelectronics^©^, Indiana, United States) with a 5 Hz GPS/GNSS module. The IMU was attached to the kayak using Velcro on the midline of the longitudinal axis of the boat, 0.15 m posterior to the kayak’s cockpit. The IMU contained a tri axial accelerometer measuring acceleration at ±2 g over a full-scale dynamic range. Accelerometer data were sampled at 50 Hz and peak detection algorithms were used to calculate SR.

#### 2.1.3 Data analysis

Power output data were obtained during ten stroke cycles (i.e., five strokes on the left side and five strokes on the right side) while paddling at the trial’s intended SR. Mean power output was subdivided into three groups: the mean power of ten strokes, and the mean power of five left and five right strokes separately. Mean kayak velocity was calculated by averaging the kayak’s velocity in the forward direction between the catch of the first stroke to the catch of the 11th stroke.

#### 2.1.4 Statistical analysis

The mean stroke power as a function of mean kayak velocity was used to establish the construct validity of the OGL paddle. Based on Equation 2, a cubic regression between mean power output and mean velocity and a y intercept of 0 was calculated for all ten strokes and the left and right strokes separately. A coefficient of determination (*R* ^2^) was used to determine the goodness of fit for each linear regression (Chicco et al., 2021). Statistical analyses were conducted in GraphPad Prism software (v.9.1.0, GraphPad Software, San Diego, United States).

### 2.2 Concurrent validity

#### 2.2.1 Paddle calibration procedure

The OGL power meter with Brača IV (765) blades was set up according to manufacturer’s guidelines (i.e., zero offset and scale factor) and calibrated using a narrow and wide weight range. The known weights used for the narrow weight calibration were 100.6 N (10.25 kg) and 247.7 N (25.25 kg), whereas the known weights for the wide weight calibration were 51.5 N (5.25 kg) and 394.9 N (40.25 kg). These weights were chosen to represent the suggested weights from OGL (narrow eight calibration) and forces that have been recorded in sprint kayaking (Bonaiuto et al., 2020). The paddle shaft was placed horizontally on two fulcrums with one fulcrum supporting the top hand position and the other fulcrum supporting the blade centre. Weightlifting plates were suspended at the bottom hand position with a small rope and metal carabiners (mass: 0.25 kg). Measurement lengths of 0.880 m, 0.345 m, 0.240 m, 1.330 m, 0.780 m, and 2.110 m were used for the blade tip to datum, datum to datum, blade tip to blade support, blade tip to shaft support, blade tip to calibration weight, and blade tip to blade tip, respectively. The blade twist was set to 60˚ right hand twist for both validations. All measurements were recorded in the OGL web application.

#### 2.2.2 Experimental protocol

**FIGURE 2:**
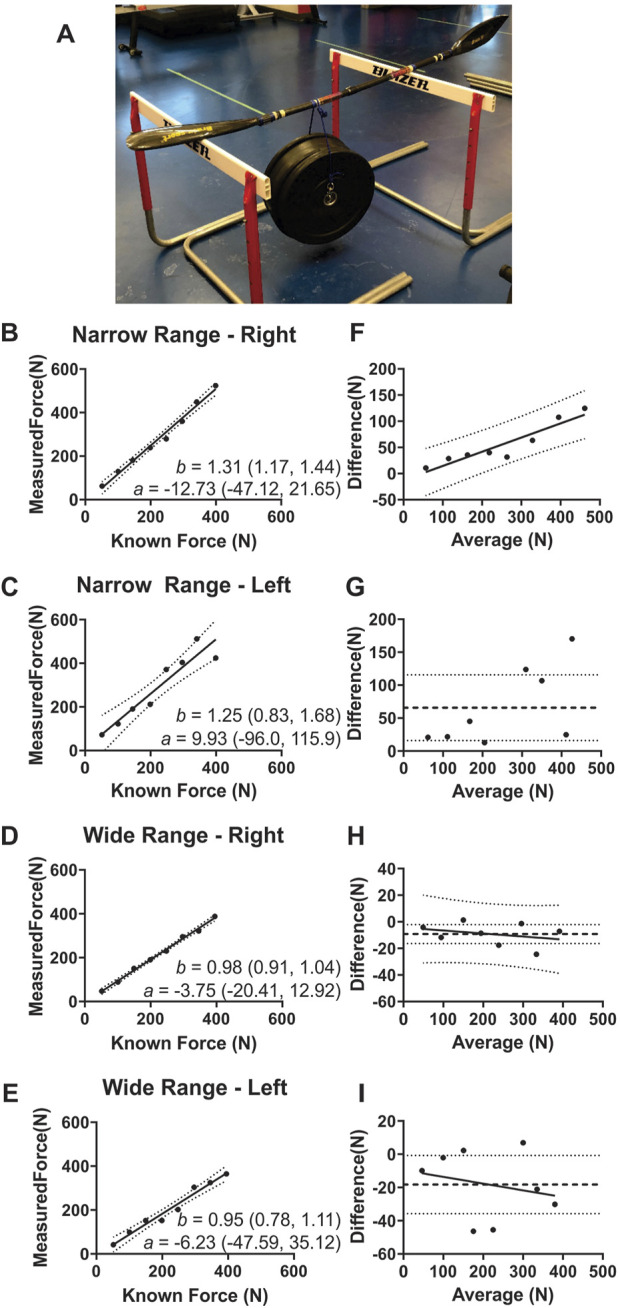
An example of the concurrent validation experimental setup. **(A)**. An example of the concurrent validation experimental setup. Linear regression data Panels **(B–E)** and Bland-Altman method of differences data Panels **(F–I)** between known forces and OGL-measured forces for the left and right shaft sides for both calibrations (narrow force range: 100.6 N–247.7 N, and wide force range: 51.5 N–394.9 N). The only calibration condition that shows a proportional bias is for the right shaft when using a narrow range of calibration Panel **(F)**. The narrow range of calibration shows a positive fixed bias Panel **(C)** whereas the wide range of calibration shows a negative fixed bias Panel **(H–I)**. a, y-intercept; b, slope; numbers in parentheses, 95% confidence intervals (CI).

Concurrent validation testing of the OGL power meter was conducted on both the right and left shaft sides, after each (narrow and wide) calibration procedures. Eight known weights (ranging from 51.5 N to 394.9 N, separated by 49.1 N increments) were hung at hand positions on both right and left shaft sides in a randomized order (Figure 2A). The weights were suspended using the same attachment system and locations used during the calibration procedure. All trials were recorded at 50 Hz and for 10 s.

#### 2.2.3 Statistical analysis

A linear regression was completed between the measured OGL paddle forces and the applied known weights (i.e., criterion measure). The linear regression’s coefficient of determination was calculated for the left and right shaft sides and calibration type. Bland Altman method of differences analyses was completed to determine if fixed and proportional bias were present in the force measurements (Ludbrook, 2010). The presence of proportional bias was determined by using an ordinary least square regression (OLS) and using an F test to determine if the slope of the method of differences data was significantly different than ‘0’. If proportional bias was present, then fixed bias was determined using an F test to establish if the y-intercept of the OLS regression between methods was different from ‘0’ (Ludbrook, 2010). If there was no proportional bias, then fixed bias was determined using a one-sample t-test comparing mean difference between methods data to ‘0’, and effect sizes were measured using partial eta squared (η2) (Ludbrook, 2010). Bland Altman analyses were conducted in GraphPad Prism. All datasets were confirmed to follow normal distributions based on D’Agostino Pearson normality tests. Statistical significance (critical α) was set at 0.05.

## 3 Results

### 3.1 Construct validity

The on-water construct validity experiment results for mean SR, velocity, force, and power output for all strokes, and left and right shaft sides are shown in Table 1. The coefficient of determination (*R* ^2^) value from the linear regression (cubic relationship: Power = x•v3) between mean paddle power and mean velocity, was 0.83 (individual range: 0.83 to 0.99; RMSE = 70.9; F_27,27_ = 70.9, p < 0.001) for all ten strokes (Figure 1B) and was 0.85 (RMSE = 68.7; F_27,27_ = 68.7, p < 0.001) for the left side of the shaft and 0.75 (RMSE = 89.4; F_27,27_ = 89.3, p < 0.001) for the right side of the shaft (Figure 1C). The coefficient value ±standard error of measurement (SEM) and the 95% confidence intervals (CI) of the combined shaft linear regression equation (x) were 6.892 ± 0.183 (CI: 6.517–7.268). The coefficient values ±SEM (and 95% CI) for the left and right shafts regressions were 7.104 ± 0.177 (CI: 6.740–7.461) and 6.681 ± 0.231 (CI: 6.207–7.154), respectively.

**TABLE 1:** Average stroke rate, velocity, force, and power outputs, and maximum force output measured in all and left and right strokes during on-water construct validation.

Variable	60 spm	80 spm	100 spm	Maximum spm
Stroke Rate (spm)	61.90 ± 1.89	83.02 ± 1.96	101.53 ± 2.78	124.04 ± 12.83
Velocity (m•s^-1^)	3.42 ± 0.14	3.89 ± 0.06	4.32 ± 0.08	4.61 ± 0.22
Mean Power All Strokes (W)	297.7 ± 57.5	416.8 ± 64.2	536.7 ± 93.7	669.4 ± 174.4
Mean Power Left Strokes (W)	313.4 ± 57.1	434.9 ± 57.8	549.1 ± 100.3	685.1 ± 181.4
Mean Power Right Strokes (W)	282.0 ± 71.5	398.6 ± 87.7	524.2 ± 105.7	653.7 ± 177.9
Mean Force All Strokes (N)	168.0 ± 51.8	185.3 ± 55.4	203.4 ± 64.2	207.0 ± 69.4
Maximum Force All Strokes (N)	270.8 ± 84.8	284.4 ± 86.9	307.7 ± 93.4	320.0 ± 101.6

Mean or maximum ± standard deviation; spm, strokes per minute; m•s^-1^; Meters per second; W, watts; N, newtons.

### 3.2 Concurrent validity

The Measured Force as a function of Known Force was represented appropriately by a linear model (Measured Force = x•Known Force + constant; Narrow-Right: F_6,6_ = 17.9, p = 0.001; Narrow-Left: F_6,6_ = 55.4, p < 0.001; Wide-Right: F_6,6_ = 8.6, p = 0.01; Wide-Left: F_6,6_ = 21.5, p = 0.001). The slopes of the linear regression from the wide weight range calibration were the closest to the optimal slope of 1, with mean slopes ±SEM (and 95% CI) of 0.98 ± 0.03 (CI: 0.91–1.04) for the right side of shaft and 0.95 ± 0.07 (CI: 0.78–1.11) for the left side of shaft (Figures 2D,E). The mean slopes ±SEM (and 95% CI) of the linear regression analyses from the narrow weight range calibration were larger (left side of shaft = 1.25 ± 0.17 (CI: 0.83–1.68); right side of shaft = 1.31 ± 0.06 (CI: 1.17–1.44) than the wide calibration (Figures 2B,C). The mean y intercept values ±SEM (and 95% CI) of the linear regression analyses were 12.73 N ± 14.05 (CI: 47.12 to 21.65) for the right side of shaft and 9.93 N ± 43.29 (CI: 96.0–115.9) for the left side of shaft for the narrow calibration, and 3.75 N ± 6.81 (CI: 20.41 to 12.92) for the right side of shaft and 6.23 N ± 16.9 (CI: 47.59 to 35.12) for the left side of shaft for the wide calibration (Figures 2B–E). The Bland-Altman method of differences identified that the narrow calibration right side of the shaft condition was the only condition to display proportional bias and the only condition to have no fixed bias (Figures 2F–I; Table 2).

**TABLE 2:** Bland-Altman method of difference results for known force vs OGL-measured force.

Force Range - Shaft	Proportional Bias				Fixed Bias				
	*r*	*b*	*P*(OLS)	Proportional Bias?	Mean Difference ±SEM (N)	95% CI (N)	*P*(t-test)	ES	Fixed Bias?
Narrow – Right	0.93	0.27	<0.001	Yes	-	−41.7, 16.6	0.397	-	No
Narrow – Left	0.66	0.28	0.076	No	65.7 ± 21.1	15.9, 115.5	0.017	0.58	Yes
Wide – Right	0.31	−0.02	0.448	No	−9.3 ± 3.0	−16.4, −2.2	0.018	0.57	Yes
Wide – Left	0.23	−0.04	0.588	No	−18.3 ± 7.4	−35.8, −0.8	0.043	0.47	Yes

Narrow force range, 100.6 N–247.7 N; wide force range, 51.5 N–394.9 N.

^a^ product-moment correlation coefficient; *b*, ordinary least squares (OLS) slope of the Bland-Altman method of differences plots; *P* (OLS), *P* value for the OLS, slope (vs 0); *P* (t-test), *P* value for the one-sample t-test on the mean differences or y-intercept (vs 0).

SEM, standard error of mean; CI, confidence interval; ES, effect size; *P* < 0.05.

Explanation of the 95% CI, column: If proportional bias is present, the 95% CI, column represents the CI, of the y-intercept. If no proportional bias is present, the 95% CI, column represents the mean difference from the Bland-Altman plotand ES, is the partial eta squared.

## 4 Discussion

This study aimed to validate the OGL power meter paddle because of its increased usage during sprint kayak training (Hogan et al., 2020a; Winchcombe et al., 2019). The results showed that the OGL power meter had both fixed and proportional bias when comparing measured forces to known forces under static loading conditions. However, only fixed bias was present when the paddle was calibrated with a wider calibration range compared to fixed bias or proportional bias when calibrating using a narrower range. Furthermore, the mean difference between the known and measured forces were approximately 3–7 times more when the paddle was calibrated with the narrow range of weights. Therefore, it can be argued that a mean error of approximately 10–20 N is small and can be used by athletes in training. As such, the calibration range should encompass the expected force ranges produced by the athletes being tested. Although the results showed the OGL paddle to have both construct and concurrent validity (when calibrated with a wide range of forces), it also showed the importance of considering the calibration procedures prior to collecting data with athletes.

The results from this study also showed that there was a strong cubic relationship between the OGL paddle’s mean power output and the athlete’s mean kayak velocity during on-water testing. This strong cubic relationship has a significant implication for elite-level sprint kayaking since for higher boat velocities, a small increment in boat velocity requires an increasingly larger increase in power. However, an important concept to consider is the construct validity does not validate the absolute power values. The construct validity results indicate that the OGL power meter results match what is expected from the cubic power-velocity relationship. Based on the concurrent validity results, if the power meter is not calibrated with an appropriate range of weights, the measured forces may have a large bias. Since power is calculated from the measured forces, the power measurements will also be biased. This concept is also relevant for the research by Macdermid and Fink (2017).

This study conducted similar concurrent and on-water construct validation protocols as Macdermid and Fink (2017). A crucial difference between studies was the inclusion of elite female sprint kayakers during the on-water construct validity assessment. For example, they showed the OGL paddle was a tool that showed construct validity for the measurement of mean power output while paddling at low kayak velocities (i.e., <2.5 m s^-1^), whereas our study showed the OGL paddle showed construct validity at higher velocities and for female sprint kayakers (i.e., between 3.42 ± 0.14 and 4.61 ± 0.22 m s^-1^) (Macdermid and Fink, 2017). Secondly, the coefficient of determination of the cubic relationships between mean power output and mean kayak velocity was greater in their study (*R* ^2^ = 0.98) compared to the current study (*R* ^2^ = 0.83). The difference between studies may be due to the number of participants tested. Our study tested seven elite female sprint kayakers, whereas their study tested a single male participant. Other factors that may have influenced the coefficient of determination differences may have been the athlete’s kayaking technique and anthropometrics. The current study is an extension of the previously published data, as the OGL paddle’s power output is now validated to velocities more appropriate to elite sprint kayakers (approximately 4.6 m s^-1^). Future research should investigate this relationship for paddling velocities reaching at least 6 m s^-1^ to include performances from male sprint kayakers (5.81 ± 0.54 m s^-1^; elite male K1 200 m sprint kayakers) (Goreham et al., 2021).

The current study and Macdermid and Fink (2017) both used static known weights to assess concurrent validity; however, the current study had eight weight trials and a larger maximal weight (394.9 N) compared to three known weights and a maximum weight of 155.9 N (Macdermid and Fink, 2017). These differences may explain why they identified a strong relative agreement between the known and measured forces with mean difference errors between 0.12% and 1.4%, while the current study identified greater absolute mean differences (Table 2) (Macdermid and Fink, 2017). In relative terms, the mean difference errors in the current study were between 0.9% and 11.7% for the right side of the shaft and 2.3%–23.4% for the left side of the shaft. Although no analytical goal was chosen for this study, multiple studies investigating other technology’s validity (e.g., IMU and GPS) have stated a mean percentage difference less than 5% is good, whereas percentages between 5% and 10% are moderate, and any value above 10% is poor (Brosnan et al., 2021; Crang et al., 2021). Again, it is suggested users of the OGL power meter calibrate their paddles with a range of forces equal to that of the kayakers they are testing.

An example of the importance of properly calibrating the OGL paddle prior to use was noticeable in a recent publication that measured bilateral force asymmetries while sprint kayaking in crew boats (Kong et al., 2020). The article presented a figure where raw force asymmetries of approximately 50–100 N were evident. The result from our study gives confidence that the OGL paddle can provide mean force differences of approximately 10–20 N under static loading conditions. By increasing the calibration force range, we saw the absolute mean difference of the left side of the shaft drop from 65.7 ± 21.1 N to 18.3 ± 7.4 N. Although no information was presented about how calibration was completed in the Kong et al. (2020) study, if they calibrated with a narrow range then their asymmetry observations may have been the biproduct of absolute mean difference errors rather than true athlete asymmetry. This further suggests the importance of internally validating equipment to ensure athlete recommendations to coaches are accurate (Brosnan et al., 2021).

### 4.1 Limitations

There were two study limitations from a statistical analysis perspective. First, an *a priori* sample size calculation was not completed, which provides the possibility of having an underpowered study that generated inappropriate estimation of the variance of the outcome variables (for example, the mean difference from the Bland-Altman method). Second, the residuals from the left shaft power measurements did not follow a normal distribution; therefore, a robust regression was also conducted on these data. The coefficient of the cubic function for the left shaft changed from 7.104 (linear regression) to 7.155 (robust regression). Since the robust regression coefficient was well within the confidence intervals of the linear regression (i.e., 6.740–7.461) the difference was not deemed to have a large effect on the overall results of the study. Finally, all participants used one OGL paddle with one set of blades, to which some athletes may not have been accustomed. However, all athletes were given ample time to warmup with the paddle before completing the trials, and no athlete stated it was difficult to paddle with the OGL paddle. Due to these reasons, we do not believe the paddle characteristics affected the results. However, by testing one OGL paddle during this experiment it introduced another limitation to the research, which was that only female athletes were studied. Due to the shorter length of the paddle, it typically only allowed for females to be tested. Although we do not expect to more differences when male sprint kayakers are tested, we have demonstrated the need to calibrate the instrument in the range of forces to be experienced. As such the range of weights for the calibration needed for male sprint kayakers may be different, but the principle remains the same.

### 4.2 Conclusion

The sport of sprint kayaking is long overdue for the power meter and force paddle technology. Currently it is common for training to be prescribed using stroke rate, distances, time, and heart rate, which are all metrics that can be affected by external factors, like the environment. By measuring power output or the paddle forces from the athlete while training the coach can determine the exact workload their athletes are enduring irrespective of the weather.

Other uses for power and force output on-water are for physical testing and development, as well as technique analysis. Strength and conditioning are a very important aspect of sprint kayaking, as all athletes are trying to increase their strength and speed capabilities while on water. Being able to measure the amount of force an athlete applies to the water, and how quickly they apply it will directly influence how their off-water training should be optimized.”

From a technical standpoint, measuring the physical output of the paddler on water allows coaches to obtain instantaneous feedback on how technique changes are influencing the athlete’s performance. This is a feature that has been missing from sprint kayaking, but is present in other sports (i.e., rowing).

In conclusion, the study establishes that even though the OGL reports power values that appear to have construct validity up to 4.6 m s^-1^, calibration with a range of weights that encompasses the projected applied forces is needed to improve the accuracy of the force measurement, and power calculation, by the OGL.

## Data Availability

The raw data supporting the conclusions of this article will be made available by the authors, without undue reservation.
